# Dermatology

**Published:** 1897-04-03

**Authors:** 


### DERMATOLOGY.

(*Continued from page 352*).

**Ichthyol.**—Dunlop,[25] in a translation of Unna's paper on this subject, draws attention to its quality in causing contraction and opposing inflamation, and points out that this quality shows in its fullest value whenever there is a normal thin epidermis or an inflammatory process of the cutis with hyperkeratosis, or finally in the parakeratosis. Under the first heading he places œdema, angiectases, urticaria, lichen, rosacea, &c.; under the second heading, eczemas, herpes, and burns; under the third heading, seborrhœa-sicca, ichthyosis, and psoriasis. He recommends it internally as a remedy for acne and for certain cases of eczema.

Chatelain[26] confirms the uses of ichthyol, proposed by Unna, and refers in addition to cases of leprosy benefited by the drug.

Schwimmer,[27] after confirming the uses of ichthyol, propounded by Unna, recommends the following preparation: Ichthyol and starch āā. 20 parts, aquæ 50 parts, with 10 parts white of egg.

McGuire[28] reports two cases of an eruption caused by ichthyol. It consisted of an inflammatory erythema with exudation, followed by an acute vesicular rash, discovered a day after the application of a mild ichthyol ointment, but which rapidly disappeared when this was stopped. In both cases the eruption was probably caused by direct contact of the ichthyol rather than by absorption through the skin, as the epidermis was unbroken.

Smith[29] discusses the chemistry and the therapeutic properties of chrysarobin and chrysophanic acid. The latter does not stain like the former, and is not irritating; but the comparative experiments made with the two substances in the treatment of psoriasis lead to the conclusion that the former is not an efficient substitute for the latter in the treatment of that disease.

**Pruritus.**—Saville[30] describes the physiology of itching, and its treatment by large doses of calcium chloride. The doses used are considerable, not less than 20 grains three times a day, and should be increased to 30 grains, to be administered after meals in a wineglass of water with tincture of orange peel to cover the salt taste. At the same time the diet should be regulated, no beer, sugar, or sweets being allowed, and meat only in moderate quantities. Although an improvement is generally noticed after the first dose, complete recovery is sometimes not obtained until the blood is saturated with the salt. It is not possible to indicate precisely which cases are most suitable for this treatment, but it is worth trying in all, where itching is a pronounced feature.

Morain[31] recommends the following treatment for pruritis of the vulva and anus. Morning and night the parts are douched with very hot water, containing 1 per cent. of chloral, carbolic acid, or vinegar, and an ointment containing menthol 45 grains, olive oil 15 minims, and lanoline 4 drachms, is applied. Instead of this ointment the following may be used: ℞. Bromide of potassium, salicylic acid (a. a.) 15 grains, glycerite of starch 5 drachms, calomel 2 grains, extract of belladona 2 grains. In other cases the following lotion may be used: ℞. Bi-chloride of mercury 30 grains, alcohol 3 drachms, rosewater 10 drachms, water a pint. In obstinate cases cauterization of the painful part, with nitrite of silver (1 drachm to the ounce), may be useful, and finally quadrilateral scarification of the skin round the anus may be necessary.

**Leprosy.**—Radcliffe Crocker[32] reports two cases of leprosy in which considerable improvement followed the hypodermic injection of perchoride of mercury; about a fifth of a grain was injected once a week, and each injection was followed by pain, which lasted about half an hour, but was not severe. He considers he has established a strong case for further trial of mercurial injections. He presumes that the modus operandi is that of a bactericide acting in the blood.

**Various.**—Payne,[33] in an address on bacteria in diseases of the skin, states that the forms most commonly met with are staphylococcus pyogenes aureus, S. albus, and more rarely the S. citreus. These organisms are often found as saprophytes on moist surfaces, *i.e.*, between the toes, under the nails, and in scurf on the scalp; they are undoubtedly the active cause of impetigo contagiosous. With regard to eczema, he is of opinion that certain clinical features of the disease may be explained as due to bacteria, in the same way as impetigo is due to staphylococci; thus it usually starts and centres where bacteria abound, and one form at least (the seborrhæic eczema of Unna) is now generally considered to be caused by micro-organisms. He has no doubt of the existence of Unna's morococcus, and of the diplococcus described by Saville as occurring in epidemic skin disease, and he states that forms are found in eczema, resembling sarcinæ, and other micro-organisms.

[25] Philadelphia, 1890. [26] Journ. de Mal. cut et syph, 1893. [27] Urin. Med. Woche. Nos. 29-30, 1892, [28] N. Y. Med. Rec., Oct. 17, 1896. [29] B. J. Derm., July, 1896, p. 267. [30] Lancet, Aug. 1, 1896, p. 300. [31] Clin. Journ., September 2, 1896, p. 304; Ther. Gaz., July 15, 1896. [32] Lancet, Aug. 8, 1896, p. 364. [33] Lancet, July 4, 1896 p. 1.

